# Localized Corrosion of CUSTOM 450 and AM 350 Stainless Steels in H_2_SO_4_ and NaCl Solutions

**DOI:** 10.3390/ma18050988

**Published:** 2025-02-24

**Authors:** Facundo Almeraya-Calderón, Miguel Villegas-Tovar, Erick Maldonado-Bandala, Maria Lara-Banda, Miguel Angel Baltazar-Zamora, Laura Landa-Ruiz, Demetrio Nieves-Mendoza, Jesus Manuel Jaquez-Muñoz, Francisco Estupiñan-Lopez, Jose Cabral-Miramontes, Javier Olguin-Coca, Citlalli Gaona-Tiburcio

**Affiliations:** 1 Universidad Autónoma de Nuevo León, FIME, Centro de Investigación e Innovación en Ingeniería Aeronáutica (CIIIA), San Nicolás de los Garza 66455, Mexico; facundo.almerayacld@uanl.edu.mx (F.A.-C.); miguel.villegastvr@uanl.edu.mx (M.V.-T.); maria.laraba@uanl.edu.mx (M.L.-B.); francisco.estupinanlp@uanl.edu.mx (F.E.-L.); jose.cabralmr@uanl.edu.mx (J.C.-M.); 2 Facultad de Ingeniería Civil, Universidad Veracruzana, Xalapa 91000, Mexico; erimaldonado@uv.mx (E.M.-B.); mbaltazar@uv.mx (M.A.B.-Z.); landar@uv.mx (L.L.-R.); dnieves@uv.mx (D.N.-M.); 3 Departamento Metal-Mecánica, Instituto Tecnológico de Ciudad Juarez, Av. Tecnológico, 1340, Ciudad Juárez 32500, Mexico; jesus.jaquezmn@uanl.edu.mx; 4 Área Académica de Ingeniería y Arquitectura, Universidad Autónoma del Estado de Hidalgo, Carretera Pachuca-Tulancingo Km. 4.5., Pachuca 42082, Mexico

**Keywords:** corrosion, cyclic potentiodynamic polarization, passivation, precipitation hardening, stainless steels

## Abstract

Precipitation-hardenable stainless steels (PHSSs) are widely used in various applications in the aerospace industry. PHSSs are used in various parts that need to combine mechanical properties with corrosion resistance when aircrafts are in corrosive environments. This study aimed to analyze the corrosion kinetics of CUSTOM 450 and AM 350 stainless steels that were passivated in acid baths for a period of 120 min at temperatures of 25 and 50 °C and then immersed in solutions containing 1 wt. % sulfuric acid (H_2_SO_4_) and 5 wt. % sodium chloride (NaCl). The electrochemical technique used was cyclic potentiodynamic polarization (CPP) based on ASTM G61-86. Microstructural characterization was performed via optical microscopy (OM) and scanning electron microscopy (SEM). The results revealed that the potentiodynamic polarization curves have two distinct corrosion mechanisms. The immersion of PHSSs in the H_2_SO_4_ solution produces an activation–passivation reaction. The NaCl solution has pseudopassivation (unstable passivation film). The values of the icorr current densities in the solutions of sodium chloride and sulfuric acids are between 10^−3^ and 10^−5^ mA/cm^2^; the stainless steels are susceptible to localized pitting corrosion in both test solutions, with positive hysteresis in the CPP.

## 1. Introduction

Various types of stainless steels are utilized in various components of the aeronautical and aerospace industries. The manufacturers of various aircraft types, such as airplanes, helicopters, jets, gliders, and the like, have repeatedly used stainless steel parts to create fully functional and safe aircraft systems [1,2]. In different industry sectors, corrosion causes significant economic losses. In the aeronautical industry, costs vary and are associated with various factors such as industry regulations, preventive maintenance practices, and technological advances [3,4,5,6,7,8].

Iron–chromium [Fe-Cr] alloys (with a minimum chromium content of 11%) with nickel additions are known as stainless steels (SSs) and have different applications. SSs can be classified based on their microstructure and precipitates as ferritic, austenitic, martensitic, duplex, and precipitation hardenable (PH) [9,10,11,12]. These steels are used in aircraft components, for example, landing gear support structures, actuators, turbine blades, fasteners, among others [6,7,8]. Stainless steels are often exposed to different corrosive environments such as industrial atmospheres (containing sulfuric acid) and coastal atmospheres.

Precipitation-hardenable (PH) steels are used in aeronautics due to their superior characteristics over other types of steels, such as their high mechanical strength, low weight, and excellent corrosion resistance, making them suitable for specific components [13,14]. PHSSs can be semi-austenitic (corrosion resistance and ductility) or martensitic (high strength-to-weight ratios). Semi-austenitic (γ and δ) stainless steel is obtained after annealing heat treatment, and martensitic stainless steel is obtained through heat treatment where the austenitic (γ) phase changes to the martensitic (α′) phase, followed by a precipitation hardening process [15,16]. The 17-4PH, 17-7PH, and 15-7Mo stainless steels most commonly used in the industrial sector were manufactured by the company Armco (USA) in 1948, followed by PHSSs AM in versions 350 and 355 and CUSTOMs 630, 455, and 450. Steels are manufactured by Carpenter Technology Corporation and ATI Materials. These steels are used to manufacture turbine blades, rotors, and shafts. For aircraft structural components, 15-5 PH steel produced by the company Penn Stainless Products, Inc. is used [17,18].

The corrosion resistance of SSs is due to the characteristic of forming a thin, invisible, compact, and protective chromium oxide film (Cr_2_O_3_) on the surface. To improve the chromium oxide film, the growth of the passivation film is carried out by the chemical process, thus providing better corrosion resistance [19,20,21,22,23]. It is therefore important to note that SSs require an essential chemical element such as chromium with contents between 17 and 18% of the ferrous alloy.

In the mid-nineteenth century, the chemist Christian Friedrich Schönbein discovered the chemical treatment called passivation, a treatment commonly performed on stainless steels through an oxidizing agent (nitric acid) [24,25,26]. Nowadays, there are currently possibilities for changing the passivation treatment, such as how to use citric acid, a biodegradable and environmentally sustainable chemical compound. The literature review indicates little information on the use and advantages of using citric acid as an oxidizing agent in the steel passivation process [27,28]. In 2003, the Boeing Company performed passivation tests on stainless steel using citric acid as an alternative for aeronautical components, and later, in 2008, the National Aeronautics and Space Administration (NASA) performed the same passivation tests, indicating a high effectiveness of this chemical treatment [21,29,30].

In recent years, research on corrosion in stainless steels has centered on pitting corrosion studies, pitting nucleation, the analysis of passive and transpassive regions, and identifying corrosion mechanisms. Corrosion measurements are based on direct and alternating current electrochemical techniques, respectively. The most commonly used electrochemical techniques are potentiodynamic polarization (PP), cyclic potentiodynamic polarization (CPP), electrochemical impedance spectroscopy (EIS), and electrochemical noise (EN). In 2014, the electrochemical noise technique was employed by Suresh and Mudali [31] where they analyze the corrosion behavior of AISI 304 SS in the presence of ferric chloride (FeCl_3_), finding an important relationship between the frequency domain through power spectral densities (PSDs) and the time domain (statistical analysis, noise resistance (Rn), and localization index (IL)) to define the localized corrosion mechanism. Lara et al. [32], in 2018, investigated the corrosion kinetics using electrochemical noise and potentiodynamic polarization in precipitation-hardened steels passivated in citric acid as an ecological alternative; the results indicated that the passivation films formed are different in the different passivation acids used. In other investigations, potentiodynamic polarization and electrochemical impedance spectroscopy were used to characterize the oxide film through charge transfer processes on passivated austenitic 304 SS; the electrochemical characteristics of SS have also been studied by varying the pH concentrations in aerated solutions, resulting in a decrease in the pH of the oxidizing solution that promotes the formation of protective films, which improve corrosion resistance [33,34,35,36,37,38].

Recent investigations on PHSSs have focused on hydrogen diffusion, fatigue behavior, and microstructural characterization, and relatively few addressed the failure analysis of components, as was the case for AM 350 steel exposed to a marine environment. Other researchers have analyzed AM 355 and martensitic steels, observing the mechanical behavior of low-cycle fatigue at different temperatures [39,40,41]. In steels such as CUSTOM 450, the machining behavior was studied using carbide tools coated with TiCN and TiAl [42,43]. Samaniego et al. and other authors [10,14,44] have studied CUSTOM 450 and AM 350 steels for the aeronautical industry using electrochemical techniques such as EN and EIS to determine the electrochemical kinetics of the passivation film in acid exhibitions. The results indicated that the martensitic steel CUSTOM 450 PHSS performed best. In 2024, Villegas et al. [9] studied CUSTOM 450 and AM 350 steels using different passivation parameters such as the time, temperature, and type of passivating bath, finding that citric acid has a better performance in the growth of the passive oxidation film for this type of SS.

The aim of this research was the localized corrosion of CUSTOM 450 and AM 350 steels which were passivated for 120 min at two temperatures (25 and 50 °C) using acid baths and then immersed in solutions containing 1% by weight of sulfuric acid and 5% by weight of sodium chloride. Cyclic potentiodynamic polarization (CPP) based on ASTM G61–86 was the electrochemical technique used. Microstructural characterization was performed via scanning electron microscopy (SEM).

## 2. Materials and Methods

### 2.1. Materials

The steels used are commercial grades such as CUSTOM 450 and AM 350 in the form of cylindrical bars. They were used and tested in the condition in which they were received. The nominal chemical composition of PHSSs is reported in the manufacturer’s technical data sheets, see Table 1.

PHSSs were prepared using the metallographic technique, using different grades (400, 500, 600, and 800) of sandpaper (silicon carbide) to roughen the surface and then polish it with a diamond paste. All samples were cleaned with deionized water and ultrasound (ethanol) for 10 min [47,48].

### 2.2. Chemical Passivation Treatment

The passivation treatment of the steels under study was carried out based on the indications in the ASTM A967-09 standard [49]. The procedure was as follows:

1. The PHSS samples were cleaned via ultrasound (10 min in ethanol) based on the ASTM A380-17 and SAE AMS 2700F standards and soaked in deionized water [50,51];
2. The passivation baths consisted of two types: citric acid (C_6_H_8_O_7_) at a concentration of 25% *w*/*v*. by weight and nitric acid (HNO_3_) at a concentration of 45% *v*/*v*;
3. The passivation solutions were at 25 and 50 °C; the immersion time was set at 120 min, ensuring adequate exposure for an effective treatment;
4. Rinsing using deionized water and air drying were performed to complete the treatment.

The description of the samples used and the nomenclature are indicated in Table 2.

### 2.3. Electrochemical Corrosion Measurement

Corrosion measurements to determine the localized corrosion of CUSTOM 450 and AM 350 steels were performed based on the electrochemical corrosion technique of CPP curves. Samples with an exposed area of 1.0 cm^2^ were exposed by immersion to two solutions: 1 wt. % H_2_SO_4_ and 5 wt. % NaCl at room temperature [52,53,54]. This type of research seeks to analyze situations where corrosive environments such as industrial and marine cause deterioration due to localized pitting corrosion. The equipment used was a potentiostat/galvanostat/ZRA (produced by Solartron 1287A, Bognor Regis, UK). The experimental setup was a typical three-electrode cell consisting of the following electrodes (E): stainless steel as the work (W), reference (R) as the saturated calomel electrode (SCE), and auxiliary (A) as the platinum mesh. The tests were performed in duplicate.

Corrosion test were performed on passivated stainless steels based on ASTM G61-86. First, the open circuit corrosion potential (E_corr_) is measured. Once the potential is stable, the CPP test uses the following parameters: scanning the potential from −1 V to +1.2 V concerning E_corr_ [54,55].

The nomenclature used to identify each of the passivated samples is as follows (time is the only parameter that is omitted from the nomenclature because it is constant).
1 or 2C or AC or N25 or 50Electrolyte H_2_SO_4_ (1) NaCl (2)Material CUSTOM 450 (C) AM 350 (A)Passivation Baths Citric Acid Nitric AcidTemperature (°C)

### 2.4. Microstructural Characterization

The microstructure of stainless steel was obtained using optical microscopy (OM, Olympus, Hamburg, Germany), and the surface (after corrosion measurements) of the passivated stainless steel was examined using a scanning electron microscope (SEM, JEOL-JSM-5610LV, Tokyo, Japan) and secondary electron (SE) detector at various temperatures. The SEM was operated with a beam energy of 20 kV, at a working distance of 8.5, and a work distance of 12 mm.

## 3. Results and Discussion

### 3.1. Microstructural Characterization of PHSSs

**Figure 1:**
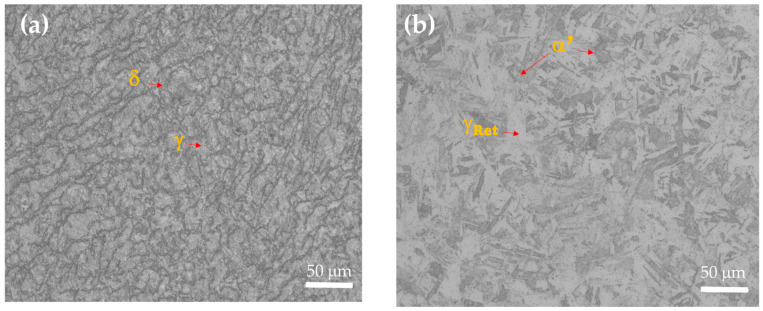
OM microstructures of PHSSs (Initial conditions): (**a**) AM 350 and (**b**) CUSTOM 450.

The microstructures of precipitation-hardenable stainless steels were obtained via scanning electron microscopy. In Figure 1a, the semi-austenitic AM 350 SS presented austenite (γ) and delta ferrite (δ) phases in its microstructure, and in Figure 1b, CUSTOM 450 stainless steel showed a martensitic phase (α′), and traces of retained austenite were present.

AM 350 is a chromium–nickel–molybdenum stainless steel that can be hardened via martensitic transformation or precipitation hardening. AM 350 may have an austenitic phase for best formability or a martensitic microstructure with high strengths depending on the heat treatment. In the annealed condition, alloy AM 350 is essentially austenitic and has forming characteristics similar to those of the AISI 300 series stainless steels. It has a higher rate of work hardening, and cold forming will cause martensite formation in proportion to the amount of deformation. It is readily hot worked from a maximum temperature of 1177 °C. The use of temperatures above this will cause an increase in the amount of ferrite. The finishing temperature should be in the 927–982 °C range to prevent grain coarsening on subsequent heat treatment and promote the homogenous precipitation of carbides. CUSTOM 450 is a martensitic stainless steel with excellent corrosion resistance and moderate strength. Although the alloy has a yield strength higher than 100 Ksi (689 MPa) in the annealed condition, it is fabricated. A single-step aging treatment causes the alloy to develop higher strength with good ductility and toughness. Mechanical properties depend on the selected aging temperature [45,46].

### 3.2. Determination of Pitting Resistance Equivalent Number

Stainless steels are susceptible to pitting corrosion due to the formation of chromium carbides that tend to precipitate at grain boundaries and cause the embrittlement of these steels. One way to quantify the susceptibility of stainless steels is through the pitting resistance equivalent number (PREN). This parameter is based on the chemical composition of this type of steel, resulting in a high PREN value, which indicates a higher resistance to pitting corrosion [56,57]. The calculation of the PREN is performed using Equation (1) based on the chromium (Cr), nitrogen (N), and molybdenum (Mo) content of the chemical composition of the stainless steel [58]. Table 3 presents the PREN results for AM 350 and CUSTOM 450 steels, where AM 350 steel has values between 25.37–29.80 and CUSTOM 450 between 17.25–20.9, respectively.

PREN = Cr + 3.3Mo + 16N(1)

### 3.3. Corrosion Measurements

Cyclic Potentiodynamic Polarization

The study of localized corrosion of CUSTOM 450 and AM 350 stainless steels was performed using cyclic potentiodynamic polarization curves, where the behavior of cathodic and anodic reactions was analyzed, and the electrochemical parameters were determined, such as corrosion current density (i_corr_), passivation current density (i_pass_), corrosion potential (E_corr_), pitting potential (E_pit_), anodic–cathodic potential (E_A-C_), and corrosion rate. The calculation of corrosion kinetics was calculated in an interval of ±300 mV in the linear section of the PP curves over at least one decade of current using the Tafel extrapolation technique [7,10,11,59,60,61,62,63,64,65].

The results obtained from this study of localized corrosion of passivated stainless steels show typical graphs where the polarization curve generated by this method will be E–log (i). A positive hysteresis loop will be observed as the potential sweep is reversed in the cyclic potentiodynamic polarization curves, indicating that the stainless steel is susceptible to localized corrosion. The susceptibility of stainless steels to localized corrosion will be expressed by the breakdown potential, or pitting potential, E_pit_, and the repassivation potential E_r_, also known as the protection potential Ep. At the pitting potential, localized corrosion begins. E_pit_ in stainless steels is often associated with the potential at which the current density suddenly increases and with the breakdown of its passive surface film, which is called transpassivation. The higher the potential (nobler), the less likely the alloy is to cause the onset of localized corrosion. At the repassivation or protection potential, pitting stops [12,16,66,67].

**Figure 2:**
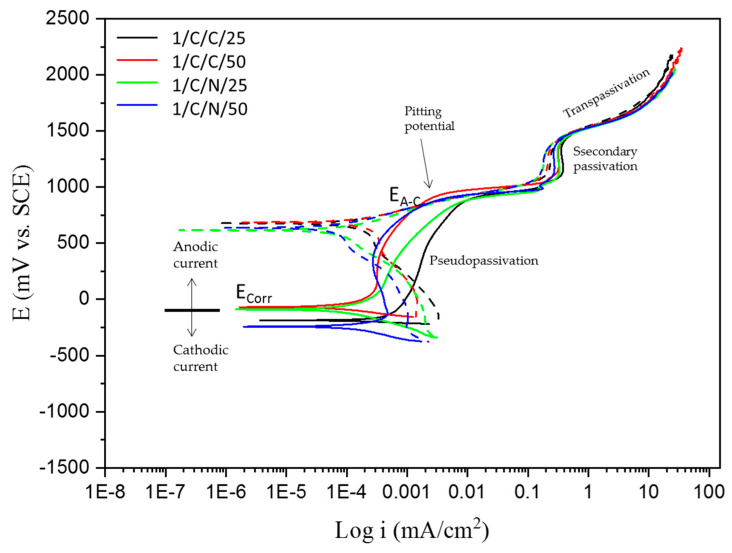
CPP curves of CUSTOM 450 samples passivated and exposed to H_2_SO_4_ solutions.

**Figure 3:**
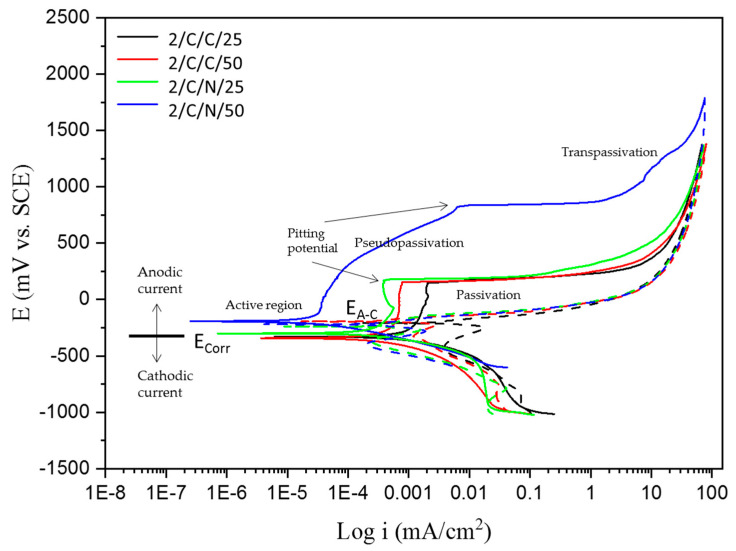
CPP curves of CUSTOM 450 samples passivated and exposed to NaCl solutions.

Corrosion behavior is seen through the cathodic and anodic regions of the CPP curves. Figure 2 and Figure 3 show the CPP obtained for the CUSTOM 450 passivated in citric and nitric acid baths at 25 and 50 °C for 120 min and immersed in the 1 wt. % H_2_SO_4_ and 5 wt. % NaCl solutions, which indicated that all systems’ anodic and cathodic reactions had mixed activation. The samples of CUSTOM 450 stainless steel (Figure 2), immersed in citric and nitric acid baths and in the H_2_SO_4_ solution, show pseudopassivation (tendency to form an oxidation film), followed by secondary passivation and having a positive hysteresis (Dashed lines on the cyclic potentiodynamic polarization curve), which indicates a susceptible to localized corrosion; the values of the current density, i_corr_, are in the order of 10^−3^ mA/cm^2^. As for CUSTOM 450 stainless steel (Figure 3), under the same passivation conditions and immersed in the NaCl solution, it presents mixed activation in the anodic and cathodic reactions; the passivation followed by transpassivation is well defined, which indicates that the oxidation layer is broken, and the return of the potentials shows a positive hysteresis with an electrochemically active area, which indicates greater susceptibility to pitting corrosion. The corrosion densities remain at 10^−4^ mA/cm^2^.

In Table 4 and Table 5, electrochemical parameters obtained are observed using CPP curves for the passivated CUSTOM 450 immersed in citric and nitric acid baths at 25 and 50 °C for 120 min and in 1 wt. % H_2_SO_4_ and 5 wt. % NaCl solutions. 

Table 4 shows the electrochemical parameters obtained by CPP of CUSTOM 450 exposed to H_2_SO_4_. The CUSTOM 450 steel samples (I/C/C/25 and I/C/N/25) in citric and nitric acids at 25 °C showed a decrease in corrosion current density from 3.8 to 1.75 (×10^−4^ mA/cm^2^), although they are in the same order of magnitude. And for the samples (I/C/C/50 and I/C/N/50) in citric and nitric acids at 50 °C, the corrosion current density increases from 1.04 to 2.7 (×10^−4^ mA/cm^2^) with the same order of magnitude. An effect of the passivation temperature can be observed in the corrosion current density values when having random values; in addition, in the cyclic potentiodynamic polarization curves, it can be observed that the CUSTOM 450 steel has zones of pseudopassivation and secondary passivation, respectively; however, all samples show positive hysteresis, indicating susceptibility to pitting corrosion. Sample I/C/C/50 presented the most noble value of samples with −0.069 V of E_corr_, indicating that the energy necessary to begin the corrosion process is higher. That result matches with the result obtained for i_corr_, with a value of 1.043 × 10^−4^ mA/cm^2^. On the other hand, 1/C/N/50 presented the more active potential with −0.242 V, indicating that the corrosion process can begin first for this sample; however, the i_corr_ is higher for 1/C/C/25, with a value of 3.837 × 10^−4^ mA/cm^2^, indicating that the corrosion kinetic is higher for this sample. The lower i_pass_ values were obtained for the passivated samples at 50 °C, meaning that passivation is more stable (a more stable passive layer) and permits the low pass of electrons. The samples passivated with citric acid showed a higher pitting potential (E_pit_) with values of 0.877 and 0.911 for 1/C/C/25 and 1/C/C/50, respectively, stipulating that pitting occurs or can be prevented at that potential. The positive value of hysteresis is related to a pitting corrosion process.

Table 5 shows the parameters for CUSTOM 450 exposed to NaCl. For the CUSTOM 450 steel samples (2/C/C/25 and 2/C/N/25) in citric and nitric acids at 25 °C, the corrosion current density shows an increase from 1.46 × 10^−4^ mA/cm^2^ to 6.494 × 10^−5^ mA/cm^2^, and for the samples (2/C/C/50 and 2/C/N/50) in citric and nitric acids at 50° C, the corrosion current density increases from 1.208 × 10^−4^ mA/cm^2^ to 9.513 × 10^−5^ mA/cm^2^. In both conditions, there is a change in the order of magnitude of the corrosion current density. The sample 2/C/N/50 presented a more noble behavior with −0.193 V of E_corr_; also, i_corr_ presents a value of 5.251 × 10^−5^ mA/cm^2^, indicating that the corrosion kinetic is lower for those samples. In contrast, 2/C/C/25 obtained an i_corr_ of 9.061 × 10^−4^ mA/cm^2^, indicating a faster corrosion kinetic. The 2/C/N/50 sample obtained a higher value of E_pit_ (0.822 V), demonstrating that the sample presented more resistance to present pitting, and the range of potential is higher. The lower i_pass_ value is for sample 2/C/N/50, with a value of 2.384 × 10^−4^ mA/cm^2^; that value is related to a more stable passive layer. All samples presented a positive hysteresis, meaning that localized corrosion occurred.

**Figure 4:**
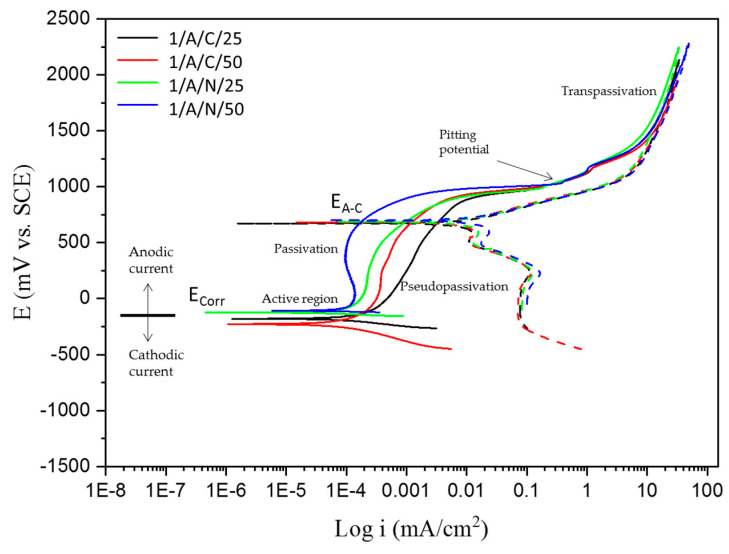
CPP curves of AM 350 samples passivated and exposed to H_2_SO_4_ solutions.

**Figure 5:**
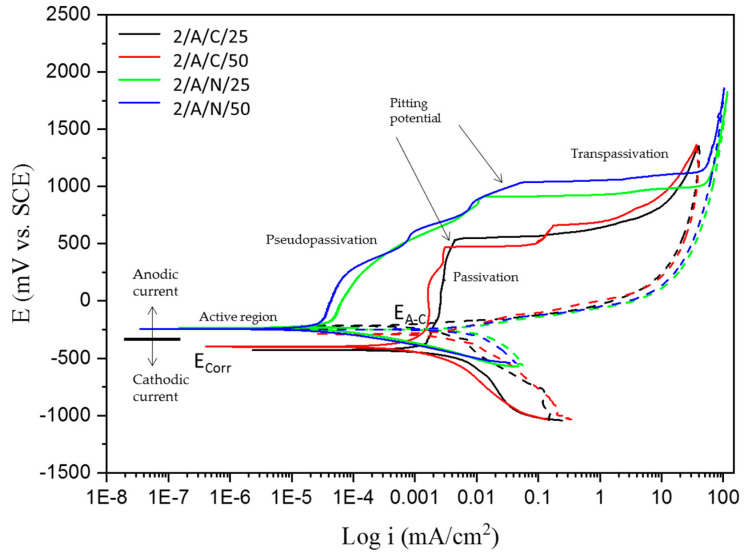
CPP curves of AM 350 samples passivated and exposed to NaCl solutions.

Regarding the results for AM 350 stainless steels passivated (Figure 4 and Figure 5) in citric and nitric acid baths at 25 and 50 °C for 120 min and immersed in 1 wt. % H_2_SO_4_ and 5 wt. % NaCl solutions, respectively, AM 350 steel shows a mixed behavior in cathodic and anodic reactions, followed by a well-defined passivation and transpassivation region, showing a greater range of passivation of the stainless steel when immersed in the NaCl solution. In the presence of sulfuric acid and sodium chloride solutions, this steel is susceptible to localized pitting corrosion by presenting positive hysteresis, with corrosion current densities of 10^−3^ to 10^−5^ mA/cm^2^, respectively.

Table 6 and Table 7 show the electrochemical parameters obtained using CPP curves for the AM 350 SS passivated in citric and nitric acid baths at 25 and 50 °C for 120 min and immersed in 1 wt. % H_2_SO_4_ and 5 wt. % NaCl solutions. Table 6 shows how 1/A/N/50 has an E_corr_ of −0.109 V, indicating a noble value compared to the other samples. Also, the i_corr_ presented has a value of 1.063 × 10^−4^ A/cm^2^, indicating that corrosion kinetic is lower than the other samples from Table 6. On the other hand, the higher corrosion kinetic is for 2/A/C/50, with a value of 7.591 × 10^−4^ mA/cm^2^.

Table 7 shows that 2/A/N/50 presented the lower corrosion kinetic, with a value of 3.422 × 10^−5^ A/cm^2^. Meanwhile, 2/A/C/25 presented the higher corrosion kinetic, with a value of 7.591 × 10^−4^ mA/cm^2^. Both tables presented positive hysteresis, indicating that localized corrosion occurred.

The cyclic potentiodynamic polarization curves of CUSTOM 450 and AM 350 stainless steels (Figure 2, Figure 3, Figure 4 and Figure 5) show a very similar trend, showing that there is passivation in the anodic reaction, except for CUSTOM 450 steel in the presence of H_2_SO_4_ which shows pseudopassivation and secondary passivation. All samples differ in pitting potential due to the different electrochemical reactions in the NaCl solution. The formation of the passive film on the surface of stainless steels determines corrosion resistance. For this reason, the corrosion protection mechanism is activated for steels containing chromium as an alloying element. Chromium oxides must fulfill the function of passive films. Their interactions with hydroxyl ions develop this protection mechanism and do not allow for an interaction with chlorine ions (Cl^−^) [62,63,67,68,69].

### 3.4. SEM Characterization of PHSSs After Electrochemical Corrosion Measurements

After performing electrochemical corrosion measurements, the surface morphology of each sample was analyzed via SEM and EDS under each condition in which they were passivated and subjected to corrosion tests. The SEM results indicate that the surface of all the passivated stainless steels presented localized pitting corrosion, with some cases showing only nucleation, while other conditions showed severe localized attacks; these results corroborate with what is indicated by the cyclic polarization curves, where all the samples presented positive hysteresis, showing susceptibility to pitting corrosion.

**Figure 6:**
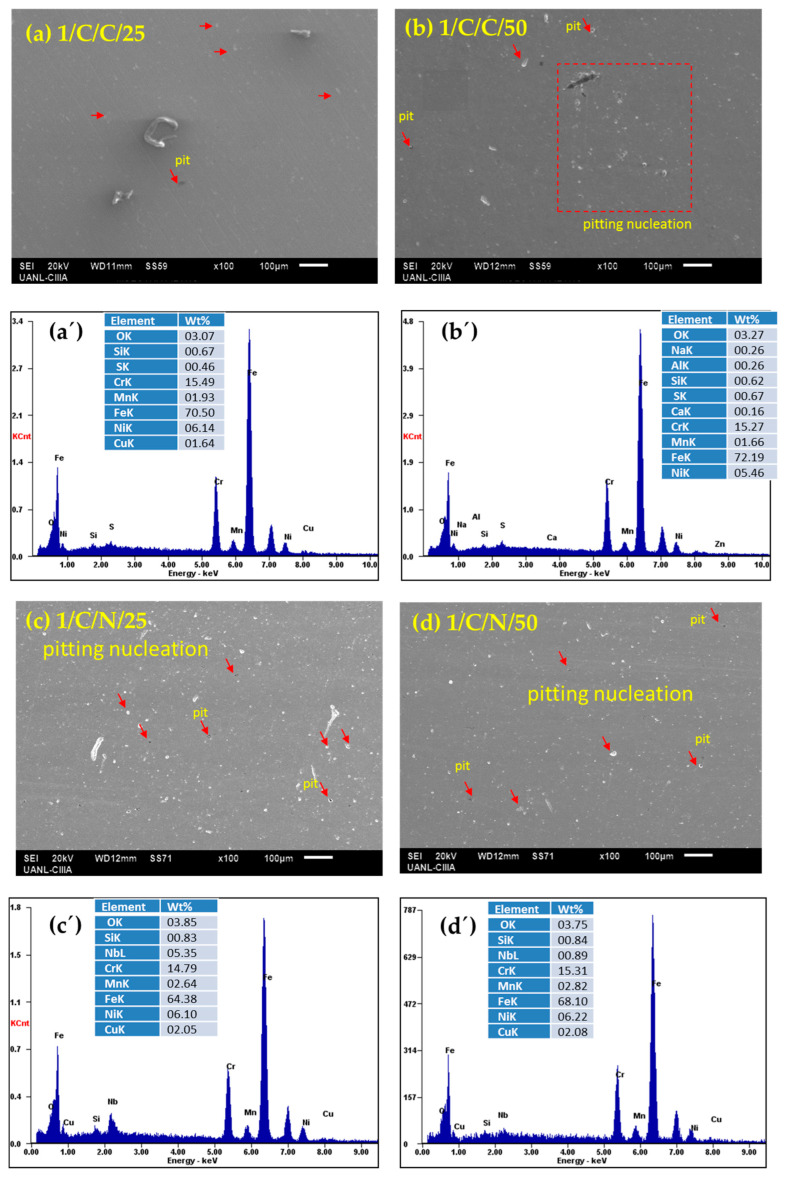
SEM-SE surface morphology of CUSTOM 450 samples passivated in citric (**a**,**b**) and nitric (**c**,**d**) acids at 25 and 50 °C for 120 min and exposed to H_2_SO_4_ solutions (after corrosion testing); (**a’**–**d’**) EDS spectrum.

The CUSTOM 450 stainless steel samples (Figure 6a–d) immersed in H_2_SO_4_, in both passivation baths and at temperatures of 25 and 50 °C, show the nucleation of pits and the presence of pits with sizes of approximately 10 microns. These results agree with the cyclic polarization curves when the hysteresis has a positive return but is practically attached to the anodic curve, which indicates that the pits are beginning to nucleate and grow. In the EDS spectra, in Figure 6a’–d’, the elemental analysis is presented, which revealed the presence of iron, chromium, manganese, and copper, which are part of the CUSTOM 450 stainless steel alloy, in addition to oxygen and sulfur from the H_2_SO_4_ solution, resulting in the nucleation of pits.

**Figure 7:**
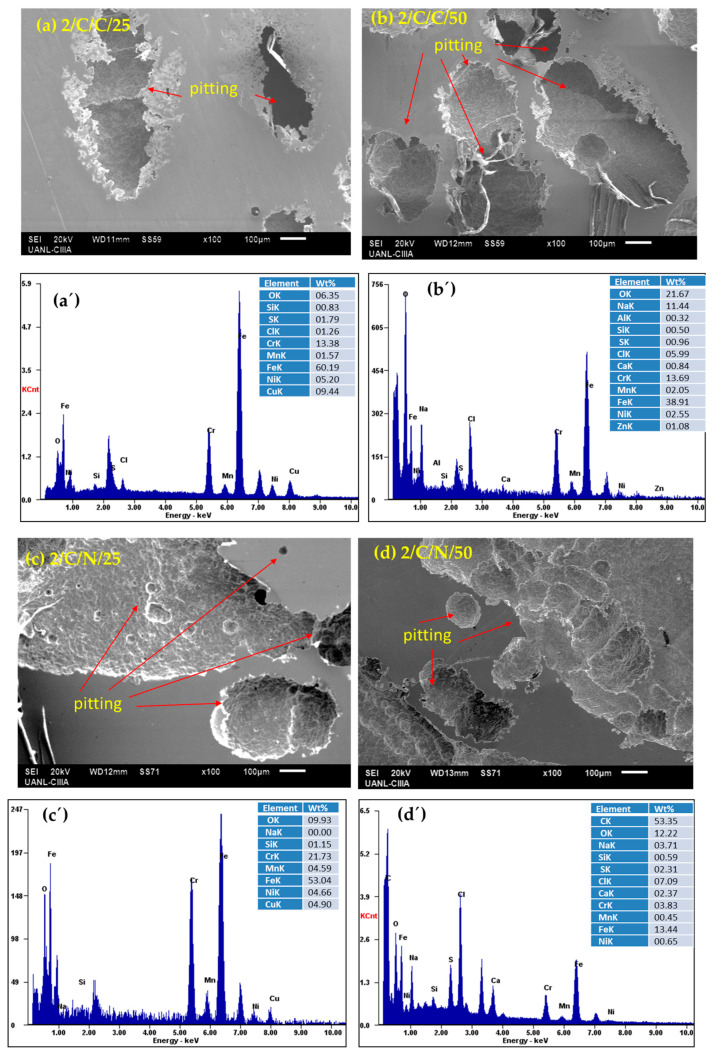
SEM-SE surface morphology of CUSTOM 450 samples passivated in citric (**a**,**b**) and nitric (**c**,**d**) acids at 25 and 50 °C for 120 min and exposed to NaCl solutions (after corrosion testing); (**a’**–**d’**) EDS spectrum.

The CUSTOM 450 stainless steel samples (Figure 7a–d), immersed in NaCl in both passivation baths and at 25 and 50 °C, exhibit pitting corrosion with sizes greater than 100 microns. In the cyclic polarization curves, the hysteresis has a positive return with a fairly large electrochemically active area, making these steels susceptible to localized pitting corrosion. In Figure 7a’–d’, the elemental analysis in the EDS spectra showed the presence of elements such as iron, chromium, manganese, and copper, which are part of the alloy, as well as elements from the sodium chloride solution. The corrosion attack is localized and much more aggressive.

**Figure 8:**
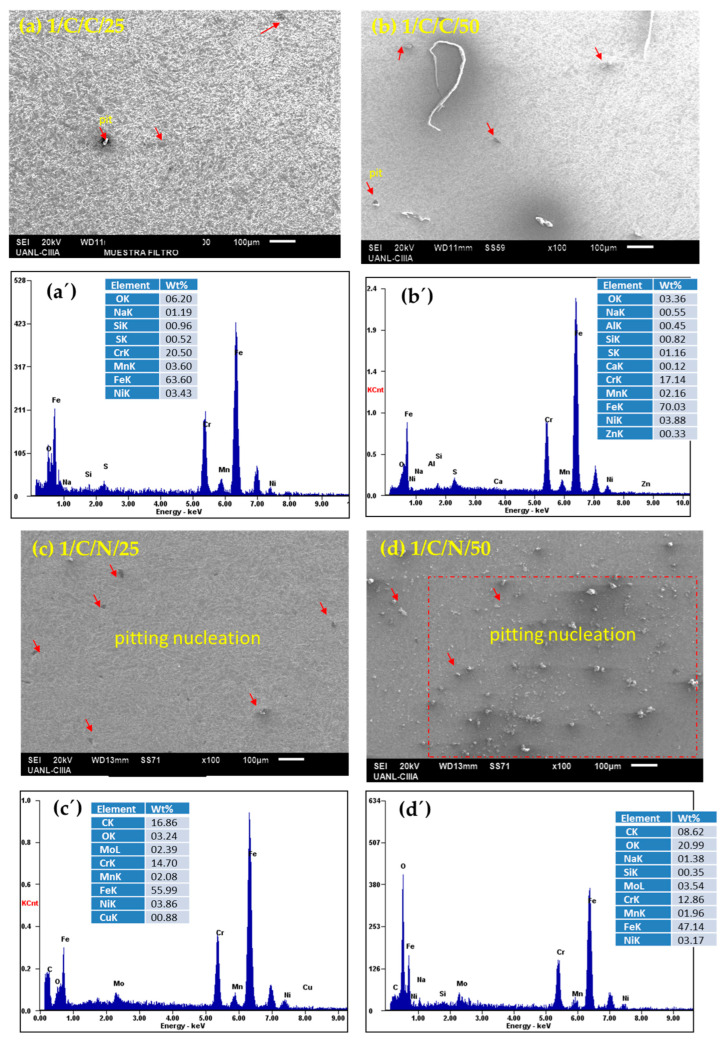
SEM-SE surface morphology of AM 350 samples passivated in citric (**a**,**b**) and nitric acids (**c**,**d**) at 25 and 50 °C for 120 min and exposed to H_2_SO_4_ solutions. (after corrosion testing); (**a’**–**d’**) EDS spectrum.

The AM 350 stainless steel samples (Figure 8a–d), immersed in H_2_SO_4_ in both passivation baths and at temperatures of 25 and 50 °C, show the nucleation of pits and the presence of pits with sizes between 10 and 30 microns. The results indicate nucleation by pitting due to the return of the positive hysteresis in the cyclic polarization curves, where there is susceptibility to pitting corrosion. In the EDS spectra in Figure 8a,b’–d’, the elemental analysis is presented, which revealed the presence of iron, chromium, and manganese, which are a part of the AM 350 stainless steel alloy, in addition to oxygen and sulfur from the H_2_SO_4_ solution. There are other trace elements, such as calcium and aluminum. The result of this steel exposed to sulfuric acid is pitting nucleation.

**Figure 9:**
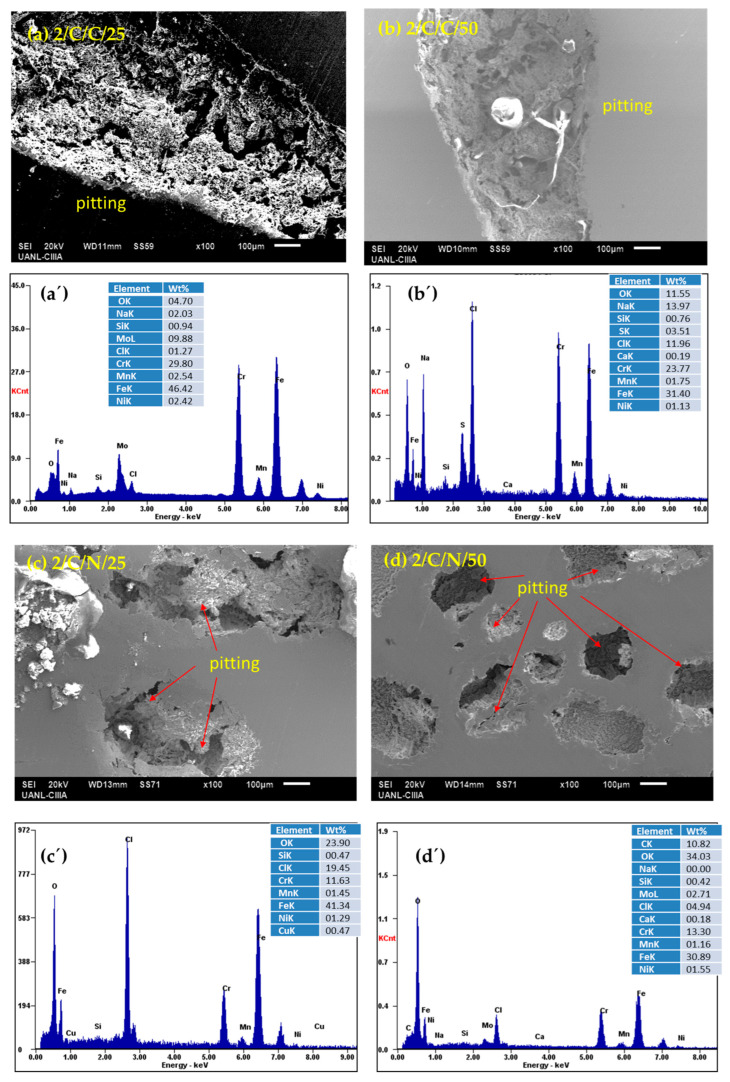
SEM-SE surface morphology of AM 350 samples passivated in citric (**a**,**b**) and nitric (**c**,**d**) acids at 25 and 50 °C for 120 min and exposed to NaCl solutions (after corrosion testing); (**a’**–**d’**) EDS spectrum.

The AM 350 stainless steel samples (Figure 9a–d), immersed in H_2_SO_4_ in both passivation baths and at temperatures of 25 and 50 °C, show pitting corrosion with pits larger than 800 microns when passivated in citric acid and pits between 100 and 200 microns when passivated in nitric acid, respectively. In the cyclic polarization curves, the hysteresis has a positive return with a large electrochemically active area, making these steels susceptible to localized pitting corrosion. Figure 9a,b’–d’ correspond to AM 350 steel, where the elemental analysis in the EDS spectra showed the presence of characteristic elements of stainless steel such as iron, chromium, and manganese, as well as elements from the sodium chloride (NaCl) solution, where the presence of Cl^−^ causes localized pitting corrosion.

Because of the different corrosion processes in the electrolytes used, the CPP curves of the PHSSs (Figure 3, Figure 4, Figure 5 and Figure 6) have different shapes, suggesting that there is passivation in the anodic reaction but with a variation in their pitting potential. Nonetheless, there are two unique reasons for the potentiodynamic polarization curves. When PHSS is immersed in the H2SO4 solution, a passivation film is formed on the surface of the chromium-rich alloy, which increases its corrosion resistance and gives rise to anticorrosive protection through passivation. Iron and chromium oxides are important in the formation of the passive film in stainless steels, since they will react with hydroxyl ions. The high i_corr_ in the PHSS samples causes transpassivation and secondary passivation. Samples immersed in the NaCl solution exhibit pseudopassivation, which is a representation of an unstable passivation layer. The base material is protected against corrosive ions such as Cl^−^ to which the samples were exposed by this defensive mechanism, which forms a passive layer of Cr-rich oxides and oxyhydroxides that prevents oxygen from entering the inner layer [7,70,71,72].

Some authors [73,74] claim that an unstable passivation coating raises the current density when samples are immersed in a NaCl solution, which alters the kinetics of stainless steel corrosion. Conversely, austenitic stainless steels immersed in a H_2_SO_4_ solution showed transients associated with transpassivation, the breakage of the passivation film, and secondary passivation, the regeneration of the passive layer.

The passive zone is where iron oxide and chromium oxide coatings, commonly detected in PHSSs, are generated [28,75,76,77]. As a result, the selective dissolution of Cr^3+^ on the surface of stainless steels produces the chromium trihydroxide complex Cr(OH)_3_ (see Equation (2)). The hydroxides react to produce a continuous passive layer of chromium oxide Cr_2_O_3_ when Cr(OH)_3_ is present on the surface (see Equation (3)) [76,77].

(2) $${Cr}^{3+}+3{OH}^{-}\to {Cr(OH)}_{3}+3e^{-}$$

(3) $$Cr{\left(OH\right)}_{3}+Cr+{3OH}^{-}\to {Cr}_{2}O_{3}+3H_{2}O+3e^{-}$$

As mentioned before, iron and chromium oxidation mainly cause anodic reactions during the passivation film development stage. The iron oxidation processes are illustrated in Equations (4)–(6) [78,79]:

(4) $$3Fe+8{OH}^{-}\to {Fe}_{3}O_{4}+4H_{2}O+8e^{-}$$

(5) $$2{Fe}_{3}O_{4}+2OH^{-}+2H_{2}O\to 6FeOOH+{2e}^{-}$$

(6) $$2{Fe}_{3}O_{4}+2{OH}^{-}\to 3{Fe}_{2}O_{3}+H_{2}O+2e^{-}$$

Only the CUSTOM 450 and AM 350 stainless steel samples, immersed in sulfuric acid for both passivation baths, present pseudopassivation, indicating an unstable oxide film and well-defined secondary passivation. Some authors indicate that the formation of the Cr(OH)_3_ film may be related to pseudopassivation [70,80]. Various investigations indicate that [62,63,64] the use of citric acid can be an ecological alternative that would allow for the replacement of nitric acid, a chemical substance that is commonly used as an anti-corrosion treatment. It is important to take into account that changing some parameters (time, temperature, and electrolytic baths) of the passivation treatment will improve the characteristics of the passive layer. It is important to note that during the dissolution of iron (anodic reactions), the oxidized films of chromium and iron form the characteristic passive film on stainless steels, which provides protection against corrosion [80,81].

It has been reported in the literature that stainless steels form a protective passive film due to the enrichment of species such as oxides and hydroxides, unlike the film formed under real ambient conditions. During chemical passivation treatment, the passive film formed is usually removed from surface contaminants, and the surface oxidation of stainless steels has a more protective passive film [80,81,82,83,84,85].

## 4. Conclusions

The present research investigates the passive state of CUSTOM 450 and AM 350 in citric and nitric acid baths at 25 and 50 °C for 120 min and when immersed in 1 wt. % H_2_SO_4_ and 5 wt. % NaCl solutions. Considering the current results of the experiments, the following can be concluded:

- AM 350 steel (25.37–29.80) showed superior corrosion resistance to CUSTOM 450 (17.25–20.9) according to PREN;
- The results of CPP, stainless steels passivated in acid baths, indicated corrosion current density values in the order of 10^−3^ to 10^−5^ mA/cm^2^;
- The electrochemical corrosion measurement (CPP) results indicate that the samples exposed to H_2_SO_4_ have pseudopassivation and secondary passivation, and all passivated SS samples exposed to H_2_SO_4_ and NaCl solutions exhibit positive hysteresis, representing susceptibility to localized pitting corrosion;
- Surface morphologies obtained via SEM indicate that the CUSTOM 450 and AM 350 stainless steel samples exhibit pitting corrosion, which is more intense when the steels are exposed to the NaCl solution due to the aggressivity of Cl^−^ ions;
- The CUSTOM 450 passivated at 25 and 50 °C for 120 min in acid baths presented the best performance, according to the results of CPP and SEM;
- The citric acid passivation treatment on SS could be an environmentally friendly alternative to the frequently used nitric acid passivation treatment.

## Figures and Tables

**Table 1:** Chemical analysis of CUSTOM 450 and AM 350 stainless steels (wt. %) [45,46].

Material	Stainless Steel Alloys											
	C	Cr	Ni	Mo	Mn	N	Si	S	Cu	Ti	Nb	Fe
CUSTOM 450	≤0.05	14.0–16.0	5.0–7.0	0.50–1.0	1.00	≤0.1	1.00	0.030	1.25–1.75	0.90–1.40	0.5–0.75	Balance
AM 350	0.07–0.11	16.0–17.0	4.0–5.0	2.50–3.25	0.50–1.25	0.07–0.13	≤0.50	0.030	–	–	–	Balance

**Table 2:** Nomenclature of the CUSTOM 450 and AM 350 SS samples.

Electrolyte	Material	Temperature (°C)	Time (min)	Passivation Baths	Nomenclature
					
H_2_SO_4_ (1)	CUSTOM 450	25	120	Citric Acid C_6_H_8_O_7_ (25% *w*/*v*)	1/C/C/25
	CUSTOM 450	50			1/C/C/50
	AM 350	25			1/A/C/25
	AM 350	50			1/A/C/50
	CUSTOM 450	25	120	Nitric Acid HNO_3_ (45% *v*/*v*)	1/C/N/25
	CUSTOM 450	50			1/C/N/50
	AM 350	25			1/A/N/25
	AM 350	50			1/A/N/50
**Electrolyte**	**Material**	**Temperature (°C)**	**Time (min)**	**Passivation Baths**	**Nomenclature**
NaCl (2)	CUSTOM 450	25	120	Citric Acid C_6_H_8_O_7_ (25% *w*/*v*)	2/C/C/25
	CUSTOM 450	50			2/C/C/50
	AM 350	25			2/A/C/25
	AM 350	50			2/A/C/50
	CUSTOM 450	25	120	Nitric Acid HNO_3_ (45% *v*/*v*)	2/C/N/25
	CUSTOM 450	50			2/C/N/50
	AM 350	25			2/A/N/25
	AM 350	50			2/A/N/50

**Table 3:** PREN of the PHSSs.

Sample	Cr	Mo	N	PREN
CUSTOM 450	14.0–16.0	0.50–1.0	≤0.1	17.25–20.9
AM 350	16.0–17.0	2.50–3.25	0.07–0.13	25.37–29.80

**Table 4:** Parameters obtained by CPP for CUSTOM 450 SS in the 1 wt. % H_2_SO_4_ solution.

Sample	E_corr_ (V)	E_A-C_ (V)	E_pit_ (V)	i_pass_ (mA/cm^2^)	i_corr_ (mA/cm^2^)	Hysteresis
1/C/C/25	−0.186	0.677	0.877	1.926 × 10^−3^	3.837 × 10^−4^	Positive
1/C/C/50	−0.069	0.686	0.911	3.193 × 10^−4^	1.043 × 10^−4^	Positive
1/C/N/25	−0.089	0.616	0.869	1.219 × 10^−3^	1.756 × 10^−4^	Positive
1/C/N/50	−0.242	0.638	0.842	3.317 × 10^−4^	2.717 × 10^−4^	Positive

**Table 5:** Parameters obtained by CPP for CUSTOM 450 SS in the 5 wt. % NaCl solution.

Sample	E_corr_ (V)	E_A-C_ (V)	E_pit_ (V)	i_pass_ (mA/cm^2^)	i_corr_ (mA/cm^2^)	Hysteresis
2/C/C/25	−0.330	−0.209	0.147	1.735 × 10^−3^	9.061 × 10^−4^	Positive
2/C/C/50	−0.342	−0.194	0.154	6.974 × 10^−4^	4.125 × 10^−4^	Positive
2/C/N/25	−0.300	−0.240	0.169	4.337 × 10^−4^	2.239 × 10^−4^	Positive
2/C/N/50	−0.193	−0.219	0.822	2.384 × 10^−4^	5.251 × 10^−5^	Positive

**Table 6:** Parameters obtained by CPP for AM 350 SS in the 1 wt. % H_2_SO_4_ solution.

Sample	E_corr_ (V)	E_A-C_ (V)	E_pit_ (V)	i_pass_ (mA/cm^2^)	i_corr_ (mA/cm^2^)	Hysteresis
1/A/C/25	−0.180	0.672	0.885	1.665 × 10^−3^	1.465 × 10^−4^	Positive
1/A/C/50	−0.228	0.683	0.881	7.262 × 10^−4^	1.208 × 10^−4^	Positive
1/A/N/25	−0.123	0.685	0.866	2.212 × 10^−4^	6.494 × 10^−5^	Positive
1/A/N/50	−0.109	0.699	0.911	1.063 × 10^−4^	9.513 × 10^−5^	Positive

**Table 7:** Parameters obtained by CPP for AM 350 SS in the 5 wt. % NaCl solution.

Sample	E_corr_ (V)	E_A-C_ (V)	E_pit_ (V)	i_pass_ (mA/cm^2^)	i_corr_ (mA/cm^2^)	Hysteresis
2/A/C/25	−0.427	−0.216	0.536	2.614 × 10^−3^	1.155 × 10^−4^	Positive
2/A/C/50	−0.399	−0.288	0.472	1.624 × 10^−3^	7.591 × 10^−4^	Positive
2/A/N/25	−0.234	−0.248	0.909	4.345 × 10^−4^	5.523 × 10^−5^	Positive
2/A/N/50	−0.245	−0.250	1.036	8.928 × 10^−4^	3.422 × 10^−5^	Positive

## Data Availability

The original contributions presented in the study are included in the article, further inquiries can be directed to the corresponding authors.
